# Ludwig's angina and airway considerations: a case report

**DOI:** 10.1186/1757-1626-1-19

**Published:** 2008-06-20

**Authors:** Anand H Kulkarni, Swarupa D Pai, Basant Bhattarai, Sumesh T Rao, M Ambareesha

**Affiliations:** 1 Department of Anesthesiology, Kasturba Medical College, Attavar, Mangalore, India

## Abstract

**Introduction:**

Patients with deep neck infections present challenging airways for an anesthesiologist. Patients with Ludwig's angina may die as a result of the inability to effectively manage the airway.

**Case presentation:**

Here we discuss the anesthetic management with fiberoptic intubation of a 45-year-old man with Ludwig's angina scheduled for emergency drainage.

**Conclusion:**

Awake fiberoptic intubation under topical anesthesia may be the ideal method to secure the airway in advanced cases of Ludwig's angina. When fiberoptic bronchoscopy is not feasible, not available or has failed, an awake tracheostomy may be the preferred option.

## Introduction

Ludwig's angina and deep neck infections are potentially lethal entities because of their tendency to cause edema, distortion, and obstruction of the airway and may arise as a consequence of airway management mishaps. In the early stages of the disease, patients may be managed with observation and intravenous antibiotics. Advanced infections, however, require the airway to be secured and surgical drainage. This is complicated by pain, trismus, airway edema, and tongue displacement creating a compromised airway. We present a recent case, successfully managed at our hospital, with a brief review of airway management options.

## Case presentation

A 45-year-old man, weighing 70 kg and 170 cm in height, presented with complaints of mouth and neck pain and difficulty in swallowing for 3 days, and had been spitting out saliva. He also reported progressive swelling in the neck and inability to open the mouth. An infected third molar had been extracted 3 days previously. He gave no history of difficulty in breathing at rest. He was nil by mouth for more than 8 hours.

**Figure 1:**
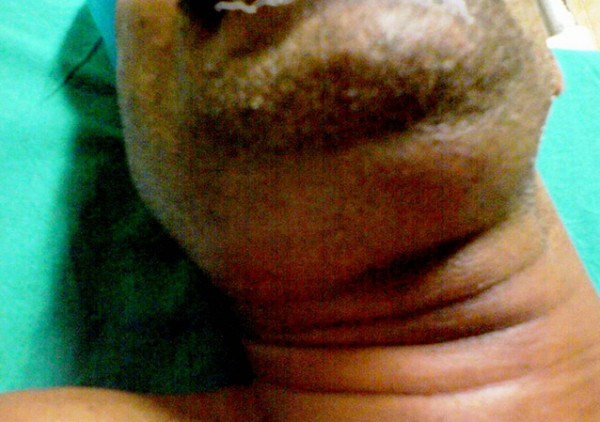
Swelling in the submandibular area. This made it difficult for the assessment of neck extension.

On physical examination, he had no respiratory distress, but was uncomfortable because of pain and intra-oral drainage of pus. He was febrile (38.8°C) with a pulse rate of 106 beats per minute, blood pressure of 140/90 mmHg and a respiratory rate of 25 breaths per minute. On airway examination mouth-opening was restricted, with an interincisor gap of 1 cm. There was a diffuse tender neck swelling, particularly in the sub-mandibular space. Neck extension was painful and limited. Both the nares were patent and the trachea was palpable in the lower part of neck (Figure 1).

A diagnosis of Ludwig's angina was made and he was scheduled for emergency drainage of the abscess. Awake fiberoptic intubation was planned, with tracheostomy as a backup. The procedure and need for awake nasal intubation was explained to the patient and written informed consent was obtained for awake intubation and tracheostomy. The patient was premedicated with intramuscular glycopyrrolate 0.4 mg. No acid aspiration prophylaxis was administered. Nasal decongestion was accomplished using oxymetazoline 0.05% nasal drops, one drop in each nostril, and lignocaine 4% topical, two drops in each nostril, was used to anesthetize the nasal mucosa. The base of the tongue and pharyngeal walls were anesthetized with lignocaine 2% viscous gargle 5 ml which was spat out, and 10% lignocaine two puffs, which was sprayed onto the posterior pharyngeal wall.

In the operating theater, electrocardiography, noninvasive blood pressure, and pulse oximetry were monitored. Intravenous access was obtained and an infusion of normal saline started. The fiberoptic bronchoscope was checked and loaded with a 6.5 mm ID cuffed endotracheal tube. We were ready to perform an emergency tracheostomy if required and the surgeon was scrubbed and ready. The fiberscope was inserted into the right nostril. We sprayed 4% lignocaine total 3 ml (spray-as-you-go) during fiberscopy, thus making the total dose of lignocaine administered 250 mg. Some difficulty was encountered as pus was draining intra-orally and periglottic edema was present. An orally placed suction catheter, however, made the fiberscopy easier. The trachea was entered and a tube was passed into the trachea. Oxygen was not administered during fiberscopy. After confirmation of tracheal intubation by fiberoptic viewing of tube tip inside the trachea, tube fogging, inability to vocalize and end-tidal carbon dioxide, anesthesia was induced with fentanyl 100 mcg and propofol 100 mg. Vecuronium 5 mg and dexamethasone 8 mg were also given. Anesthesia was continued with nitrous oxide in oxygen and halothane with closed circuit and intermittent positive pressure ventilation. Intra-operatively the vitals were stable. Incision and drainage were performed by the sub-mandibular route and a drain left behind.

At the end of the procedure residual neuromuscular blockade was antagonized using neostigmine 3 mg and glycopyrrolate 0.6 mg. In view of the periglottic edema, the trachea was not extubated, and the patient was moved to a postanesthesia care unit with the endotracheal tube *in situ *and placed on a T-piece with oxygen at a flow rate of 5 liters per minute. Sedation was attained using midazolam and fentanyl titrated to effect.

The following morning the patient was comfortable, with a pulse rate of 68 beats per minute, blood pressure of 110/70 mmHg and oxyhemoglobin saturation of 97%. The neck swelling had subsided. A thorough oral suction was performed and the trachea was extubated. The fiberscope was kept handy; however, no elaborate preparations for tracheostomy or similar procedures were made in view of the edema having subsided and as there was no significant anticipation of airway difficulty. Postextubation recovery was uneventful. The patient was discharged 4 days later.

## Discussion

While described as far back as the writings of Hippocrates and Galen, the necrotizing fasciitis Ludwig's angina was first detailed by the German surgeon Wilhelm Friedrich von Ludwig in 1836 [1]. Ludwig's angina is a rapidly progressive, potentially fulminant cellulitis involving the sub-lingual, sub-mental, and sub-mandibular spaces [2]. It typically originates from an infected or recently extracted tooth, most commonly the lower second and third molars [2]. The sub-mandibular space is involved by penetration of the thin inner cortex of the mandible by periapical dental abscesses. Spread to the sub-lingual space is around the posterior margin of mylohyoid muscle [1]. It has, however, been reported as a result of mandibular fracture, sub-mandibular sialadenitis, peritonsillar abscess, epiglottitis [3], and oral malignancies [4].

Ludwig's angina begins as a mild infection and can rapidly progress to brawny bilateral induration of the upper neck with pain, trismus, and tongue elevation. Fever and dysphagia are common. The most serious complication of Ludwig's angina is asphyxia caused by expanding edema of soft tissues of the neck [5]. Another common cause of death is the acute loss of airway during interventions to control the condition [4]. Stridor, difficulty managing secretions, anxiety, cyanosis, and sitting posture are late signs of impending airway obstruction and indicate the need for an immediate artificial airway [6]. A computed tomography scan is helpful in assessing the extent of retropharyngeal extension of the abscess [7] and may also help to decide when an artificial airway is required [8]. Other complications which may occur are the spread of infection to the mediastinum, carotid sheath, skull base, and meninges, reaching a mortality rate of 20% to 50% (see [2]).

Ludwig's angina was formerly invariably fatal but now, with adequate surgical and antibiotic treatment, has a much reduced rate of mortality [9]. It remains, however, a potentially life-threatening condition because of the risk of impending airway obstruction [10]. Thus, because of its invasive nature, early recognition and treatment of Ludwig's angina is extremely important [11].

Intravenous dexamethasone and nebulized adrenaline have been used to reduce upper airway edema in such cases to defer or avoid airway instrumentation altogether [12]. Distorted airway anatomy, tissue immobility, and limited access to the mouth make orotracheal intubation by direct laryngoscopy difficult. In advanced cases, induction of general anesthesia is dangerous because this may precipitate complete airway closure and make mask ventilation and intubation impossible [7]. Securing of the airway in the awake state is therefore the safest option.

Blind nasal intubation is to be avoided as, besides having a high failure rate, it could cause catastrophic bleeding, laryngospasm, airway edema, rupture of pus into the oral cavity, and aspiration. Complete airway obstruction could be precipitated, potentially necessitating an emergency cricothyrotomy [4]. Classically, tracheostomy was considered as the standard of care for establishment of a definitive airway [13]. Elective awake tracheostomy has been suggested for all patients with deep neck infections in order to avoid the dangers of emergency tracheostomy in a severely compromised airway [10]. This notion has since been questioned [14].

Although distorted anatomy, edema, and secretions may contribute to difficulty with fiberoptic intubation, in skilled and experienced hands, flexible fiberoptic nasal intubation is the preferred method of airway management [12] and has a high rate of success [4]. Application of topical anesthesia enables the patient to tolerate the procedure with greater comfort.

When fiberoptic bronchoscopy is not feasible, not available, or has failed, cricothyrotomy and tracheostomy are the options. Tracheostomy may be difficult or impossible in advanced cases of neck infection because of the position needed for the procedure and anatomical distortion of the anterior neck [4]. The choice of airway maneuvers must be individualized, depending on the judgment and experience of the physician in charge [5].

The causative bacteria are often Gram-negative anaerobic organisms, and penicillin, clindamycin, or ciprofloxacin are often the antibiotics of choice. In addition, surgical intervention is common, particularly in the presence of fluctuation, to incise and drain the infected space.

## Conclusion

It is important to recognize Ludwig's angina in the earlier stages of the disease, when it is easier to manage. In advanced cases, however, securing the airway and surgical drainage are important. Awake fiberoptic intubation under topical anesthesia may be the ideal method to secure the airway. When fiberoptic bronchoscopy is not feasible, not available, or has failed, an awake tracheostomy may be the preferred option.

## Competing interests

The authors declare that they have no competing interests.

## Authors' contributions

All of the authors were involved in the management of the case and finalizing the article. All of the authors were involved in the process of editing, correcting, and finalizing the manuscript. All authors have read and approved the final manuscript.

## Consent

Written informed consent was obtained from the patient for publication of this case report and accompanying images. A copy of the written consent is available for review by the Editor-in-Chief of this journal.
